# An ex vivo tissue model of cartilage degradation suggests that cartilage state can be determined from secreted key protein patterns

**DOI:** 10.1371/journal.pone.0224231

**Published:** 2019-10-21

**Authors:** Michael Neidlin, Efthymia Chantzi, George Macheras, Mats G. Gustafsson, Leonidas G. Alexopoulos

**Affiliations:** 1 Department of Mechanical Engineering, National Technical University of Athens, Athens, Greece; 2 Department of Medical Sciences, Uppsala University, Uppsala, Sweden; 3 4th Orthopaedic Department, KAT Hospital, Athens, Greece; Rush University Medical Center, UNITED STATES

## Abstract

The pathophysiology of osteoarthritis (OA) involves dysregulation of anabolic and catabolic processes associated with a broad panel of proteins that ultimately lead to cartilage degradation. An increased understanding about these protein interactions with systematic in vitro analyses may give new ideas regarding candidates for treatment of OA related cartilage degradation. Therefore, an ex vivo tissue model of cartilage degradation was established by culturing tissue explants with bacterial collagenase II. Responses of healthy and degrading cartilage were analyzed through protein abundance in tissue supernatant with a 26-multiplex protein profiling assay, after exposing the samples to a panel of 55 protein stimulations present in synovial joints of OA patients. Multivariate data analysis including exhaustive pairwise variable subset selection identified the most outstanding changes in measured protein secretions. MMP9 response to stimulation was outstandingly low in degrading cartilage and there were several protein pairs like IFNG and MMP9 that can be used for successful discrimination between degrading and healthy samples. The discovered changes in protein responses seem promising for accurate detection of degrading cartilage. The ex vivo model seems interesting for drug discovery projects related to cartilage degradation, for example when trying to uncover the unknown interactions between secreted proteins in healthy and degrading tissues.

## Introduction

Osteoarthritis (OA) is a progressive disease involving mechanical, biochemical and genetic factors that disturb associations between chondrocytes and extracellular matrix (ECM), alter cellular metabolic responses and result in degradation of articular cartilage1. Prominent proteins associated with the pathophysiology of OA are pro-inflammatory cytokines including the interleukins IL1a/b, IL6, IL8 and the tumor necrosis factor TNFa [1]. Anti-inflammatory cytokines such as IL4, IL10 and IL13 are also elevated in OA tissues [2]. Moreover, aggrecanases and matrix metalloproteinases (MMPs) that degrade the ECM as well as growth factor families of bone morphogenetic proteins (BMPs), fibroblast growth factors (FGFs) and transforming growth factors (TGFs) are all present in synovial joints of OA patients [3,4]. The fact that proteins with opposing effects are found in OA joints simultaneously (e.g. pro-inflammatory and anti-inflammatory cytokines or matrix degrading enzymes and chondrogenic cytokines) suggests nontrivial inherent interactions between these proteins.

Targeting these players separately in order to reverse or suppress OA has been tested in many clinical studies in the past with very limited success. Monoclonal antibodies such as Adalimumab (anti-TNFa) and Canakinumab (anti-ILb), MMP inhibitors, and growth factor stimulators like Sprifermin (rhFGF18) have not been able to provide significant improvements until now or are still in clinical trials [5]. Such rather disappointing results support the hypothesis that targeting a single protein is not sufficient for a successful therapy. Therefore, a deeper understanding of the cytokine interaction network might be necessary to leverage drug discovery in OA.

One approach to achieve this aim is the use of antibody-based multiplexing assays that simultaneously measure the abundance of a broad panel of proteins in a biological sample. Applications of such assays related to OA and cartilage include reconstructions of chondrocyte cell signaling pathways based on phosphoproteomics and cytokine release data from 2D chondrocyte cultures [6], cytokine releases after anabolic stimulations of 3D chondrocyte scaffolds [7] and measurements of joint pathology dependent cytokine profiles in synovial fluids and cartilage tissues [8]. Notably, careful multivariate analyses of such protein measurements also have great potential as a tool for discovery of novel diagnostic biomarkers in the form of characteristic changes across subsets of the proteins studied. However, in order to enable systematic large-scale measurements of these protein secretion patterns, a sufficiently simple and cheap ex vivo tissue model of cartilage degradation (CD) is needed.

Many in vitro models of OA and CD have been developed in the past [9]. These differ in the tissue types used (monolayer cell cultures, 3D cell cultures or tissue explants) and the method chosen for OA induction (mechanical damage or chemical stimulation with pro-inflammatory cytokines). Some in vitro models use co-culturing with synovium, subchondral bone or other OA related tissues to represent more physiological conditions. Chemical induction of OA in an explant model often uses IL1b and/or TNFa to suppress the synthesis of proteoglycans and increase the release of MMPs that consequently cleave the collagen links of the ECM [9]. Another approach for modeling of CD associated with OA, recently proposed by Grenier et al. [10], is pre-treatment of cartilage tissue explants by collagenase type II. Using this approach, cleavage of collagen II is directly induced and the ECM gets degraded, together with associated changes in surface morphology, decreases of tissue sulfated glycosoaminoglycan (s-GAG) content and a deterioration of the mechanical properties such as increased permeability and decreased Young’s moduli. As stated by the Grenier et al. [10], this suggests that enzymatic degradation with collagenase II can be used to simulate characteristic changes observed in early-stage OA.

Similarly to Grenier et al. [10] we therefore used pure collagenase II pre-treatment as a degradation inducer in order to create a simplified ex vivo tissue model of CD. Using only collagenase II results in an oversimplification of the physiological conditions, but one can be sure that degradation will be achieved after a rather short treatment period. This model was established and then used in our protein profiling approach to understand how different protein stimuli affect the degraded state and to search for potential diagnostic biomarkers that can discriminate between healthy and degraded cartilage tissue. More specifically, we expanded the work by Grenier et al. [10] by looking at protein secretion patterns after stimulation with major OA related cytokines/proteins, and evaluated the possibility to use these response patterns for determination of the cartilage state. As demonstrated below, this novel systematic approach revealed biomarkers with potential to be used for accurate detection/diagnostics of degrading cartilage. More generally, this approach was found to have potential to help uncovering the interactions of CD related proteins, and thereby also help accelerating drug discovery and development activities associated with OA.

## Materials and methods

### Explant tissue model

#### Model overview and workflow

The main idea of the ex vivo tissue model is to perturb healthy and degrading cartilage tissue with a set of OA related stimuli followed by a measurement of the tissue responses in terms of protein secretions. The resulting dataset is analyzed in order to compare the two different tissue states and pinpoint individual stimuli yielding different protein responses. Our hypothesis is that these protein responses depend on the tissue state (healthy or degrading) and thus can be used to distinguish between them.

**Fig 1:**
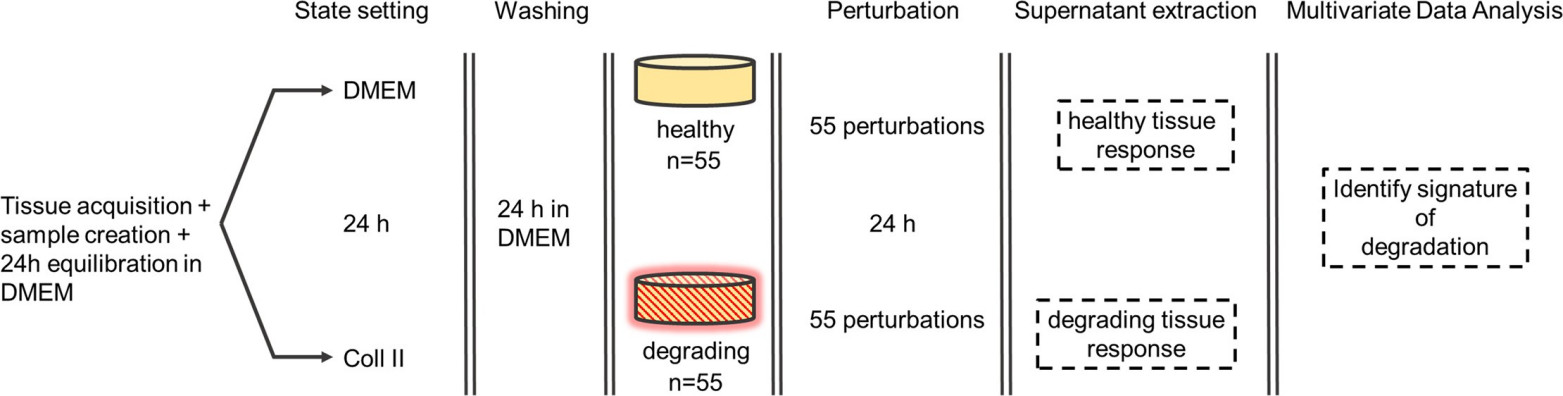
Overview of the combined ex vivo and in silico procedure. Cartilage tissue explants were retrieved from femoral heads of hip fracture patients and equilibrated for 24h in DMEM*. Tissue discs were pre-treated for 24h either with DMEM* or collagenase type II, and washed with DMEM* for 24h. Then the discs were stimulated for another 24h with 55 perturbations consisting of single proteins and pairwise combinations of some of them. Healthy and degrading responses were characterized in terms of multivariate patterns of secreted proteins measured in the supernatant after the stimulation.

Fig 1 illustrates the combined experimental-computational procedure with the individual steps described in more detail below.

#### Explant isolation, state setting and washing

Cartilage tissue samples were obtained from the femoral heads of patients (n = 4, age 72–82, 2 male and 2 females) undergoing total hip replacement due to fracture with patient’s informed consent and protocol approved by the responsible ethics committee of the KAT General Hospital. The samples were examined macroscopically to determine the locations of intact cartilage and almost all parts of the femur head could be used. Femur heads were rinsed with PBS, cartilage without subchondral bone was removed and placed into high glucose DMEM (Dulbecco's Modified Eagle Medium) supplemented with 10% FBS, 1% Penicillin/Streptomycin and 1% fungizone (BioCell Technology LLC, Irvine, CA), denoted by DMEM*. Cartilage disc samples of 3mm diameter were created with a biopsy punch and let to equilibrate in DMEM* for 24h. Then the tissue samples were placed in either fresh DMEM* or DMEM* with collagenase type II, activity 125 units/mg, (MP Biomedicals, Santa Ana, CA) of 2 mg/ml for 24h. To see the effect of different collagenase concentrations on the cytokine/protein secretion of the cartilage explants, concentrations of 1 and 4 mg/ml were also used. The three collagenase concentrations were applied in duplicate. Finally, before starting the perturbations of the resulting healthy and degrading cartilage samples, a washing step of 24h in fresh DMEM* media was included.

#### Perturbation and supernatant extraction

A total number of 13 proteins for stimulation were selected as they have been reported being present in OA [1,2]. These included IL1a, IL1b, TNFa, IL6, IL8, IL4, IL10, IL13, BMP2, FGF2, IGF1, TGFb1 and MMP9 (PeproTech EC Ltd, London). These proteins were separated into the following two groups; [IL1a, IL1b, TNFa, IL6, IL8, MMP9] and [IL4, IL10, IL13, BMP2, FGF2, IGF1, TGFb1]. All possible single treatments and all possible pairwise combinations from these two groups were used as a perturbation set, resulting in 55 different stimuli. Thus, the first group included proteins with mainly pro-inflammatory (IL1a, IL1b, TNFa), ambivalent activity (IL6 and IL8) and matrix degrading/catabolic activity (MMP9). The second group consisted of proteins with mainly anti-inflammatory (IL4, IL10, IL13) and chondrogenic/anabolic activity (BMP2, FGF2, IGF1, TGFb1). The concentrations used were chosen from prior experiments and already existing studies [6,7,9]. Detailed information on the experimental design can be found in S1 File. A stimulation time of 24h was chosen based on studies about the transient behavior of tissue responses after stimulation duration of 6h, 24h, 48h and 72h. The detailed analysis is in S2 File. In S3 File the results show that no strong batch effect due to patient-to-patient variability could be observed and also that the coefficient of variation of the assay was acceptable (<25%) for 24 out of 26 proteins. Cartilage discs were put in 96 well microplates and individually stimulated with 260 μl of media. After the stimulation 80μl of the supernatant was retrieved and cytokine releases were measured with the FlexMap 3D platform (Luminex Corp. USA). The supernatant of an unstimulated disc (cultured in DMEM* during the perturbation step) was taken as control. Blank measurements to evaluate the experimental noise for each protein were included as well. All steps were conducted in a humidified incubator at 37°C and 5% CO2. The rather high number of stimuli reduced the possibility of having biological replicates as 55 cartilage explants were needed for one run of single measurements. As our main aim was to discover and compare response patterns of cartilage explants and not to uncover new biological mechanisms, we decided to accept the drawback of having a low number of replicates with simultaneously having a broad panel of stimuli. Thus, single measurements were collected after the stimulations of 55 untreated cartilage discs of patient P2 and 55 collagenase II (2 mg/ml) treated cartilage discs of patient P3.

### Experimental techniques

#### Multiplex ELISA

The Luminex xMAP technology is an antibody-based suspension array technology measuring protein abundance in a sample for a set of predetermined proteins. Detailed background information can be found in the review of Alexopoulos et al.[11]

A library of 26 protein releases (PEDF, CXCL11, IL13, ZG16, IL4, GROA, IFNG, CYTC, IL8, IL17F, IL12A, TNFa, IL1a, TFF3, ICAM1, IL10, FST, S100A6, CXCL10, PROK1, CCL5, IL20, TNFSF12, BMP2, FGF2, MMP9) was measured in the supernatant.

#### Histology evaluation

DMEM* and collagenase II (2 mg/ml) treated cartilage discs were taken after the washing step (Fig 1), fixated in PBS with 10% formalin, decalcified and embedded in OCT. Histological evaluation with toluidine blue staining following standard protocols was performed [12].

#### Mechanical testing

In order to evaluate the change of mechanical properties after collagenase II treatment, five DMEM* and five collagenase II (2mg/ml) treated tissue samples were taken after the washing step and tested with the Bose Electroforce 3100 (Bose, Framingham, MA). Stress-relaxation tests with subsequent calculation of material parameters was used to obtain information about the material properties [13]. Initially, samples were pre-loaded with a force of F = 0.1N. Then an instantaneous ramp displacement of 5% of the initial height was applied and the relaxation of the force over time was measured until a dynamical equilibrium was reached. The procedure was repeated for a total of three loading steps. Material parameters were identified with a finite element modeling approach as described below.

#### GAG release

The extracellular release of sulfated glycosaminoglycans was measured spectrophotometrically via a Dimethylmethylene Blue (DMMB) assay [14] using the Varioscan LUX multimode microplate reader (Thermofisher Scientific Inc., USA). As s-GAGs belong to the main constituents of the ECM [10] an increased presence in the explant supernatant can be directly related to increased ECM destruction. The GAG release was quantified for the DMEM* and the collagenase II (2 mg/ml) groups. 50 μl of the supernatant was extracted after 24h of stimulation. The measured GAG concentration was normalized to tissue wet weight. 5 discs were chosen per group.

#### Collagen II content

Collagen II is the main constituent of the ECM [10], thus reduction can be directly related to ECM destruction. Tissue collagen II content was quantified as shown previously [15] with a hydroxyproline assay kit (Abcam, Cambridge, UK). One group was treated with DMEM* for 24h and the other group was treated with collagenase II (2 mg/ml) for 24h. 5 discs were chosen per group and tissue collagen content was normalized to tissue wet weight.

### Data post-processing, analysis and statistical tests

The multiplex ELISA experiments delivered median raw fluorescence intensities (MFIs) for each marker in the cytokine release dataset resulting in (55 perturbation +1 control)*26 (proteins) = 1456 data points for the healthy and degrading tissue, respectively. Additionally duplicate blank measurements were included for each protein. MFI values of measurements below the average of the blanks were deleted and imputed based on a nearest neighbor algorithm [16] as implemented in the function *knnimpute* provided by Matlab (MathWorks, Natick, USA). In addition, the same imputation algorithm was used to replace the saturated MFI values obtained for the particular protein(s) used for stimulation. For example, when IL1a is used for stimulation, then the corresponding MFI value is saturated and thus replaced via imputation.

Meaningful multivariate data analyses required normalized MFIs that allow comparisons between plates and between cytokines/proteins. The normalized difference *D(i*,*j*,*p)* for stimulus *i* and cytokine/protein *j* present on plate *p* was determined in Eq (1):

$$D(i,j,p)=\frac{(F(i,j,p)-F_{c}(j,p))/2}{(F(i,j,p)+F_{c}(j,p))/2}=\frac{F(i,j,p)-F_{c}(j,p)}{F(i,j,p)+F_{c}(j,p)}$$ (1)

where *F(i*,*j*,*p)* denotes the MFI from cytokine *j* for stimulus *i* on plate *p* and *F*_*c*_*(j*,*p)* denotes the signal from the untreated control well for cytokine *j* on plate *p*. The normalized values are all restricted to the interval [-1,+1] where the value +1 is obtained when *F(i*,*j*,*p)>>F*_*c*_*(j*,*p)*, the value -1 when *F(i*,*j*,*p)<<F*_*c*_*(j*,*p)* and the value 0 when *F(i*,*j*,*p) = F*_*c*_*(j*,*p)*. The normalized data is included in S1 Table. Matlab and R [17] were used for the post processing and the analysis of the data. Principal Component Analysis [18] (PCA) and k-means clustering [19] were used to explore the data. Subsequently, supervised methods in the form of optimal orthogonal system discriminant analysis (OOS-DA) [20] and an exhaustive pairwise variable subset selection procedure were employed in order to identify the most discriminative pairs of proteins.

#### OOS-DA, optimal orthonormal system for discriminant analysis

OOS-DA [20] may be viewed as a multi-output generalization of Fisher’s linear discriminant analysis [21] where multiple orthogonal linear projections are created sequentially, each maximizing Fisher´s separation criterion between the classes of interest. OOS-DA has been used previously to design a new type of classification method [22] and for batch correction in mass spectrometry based metabolomics [23]. The OOS-DA was performed by means of Matlab code developed in-house.

#### Exhaustive pairwise variable subset selection

Since there are many linear combinations of the protein levels that can result in complete separation of degraded and healthy cells, we investigated also the possibility of finding pairs of proteins that can offer good separation as follows. First a separation score *J*_*separation*_ was defined as:

$$J_{separation}=\frac{s_{b}}{s_{w}}$$ (2)

where s_b_ quantifies the spread between the two classes (stable and degraded) and s_w_ quantifies the spread within the two classes. More specifically, the two quantities are defined as:

$$s_{w}=\sum_{k=1}^{K}\frac{1}{N_{k}}\sum_{n\in S\left(k\right)}{\left|\left|x_{n}-m_{k}\right|\right|}^{2}\;\mathrm{where}\;m_{k}=\frac{1}{N_{k}}\sum_{n\in S\left(k\right)}x_{n}$$ (3)

$$s_{b}=\frac{1}{K}\sum_{k=1}^{K}{\left|\left|m_{k}-m\right|\right|}^{2}\;\mathrm{where}\;m=\frac{1}{K}\sum_{k=1}^{K}m_{k}$$ (4)

Here ***x***_*n*_ denotes the nth sample (column vector), *S(k)* denotes the set of samples belonging to cluster k, and *N*_*k*_ denotes the number of samples belonging to class *k*. Then in the analysis performed, we used *K = 2* classes corresponding to healthy and degraded samples, respectively. In order to reduce the risk of overfitting, the actual score used to identify the most discriminating protein pair was calculated as the maximum across 100 values of *J*_*separation*_ obtained using a resampling approach where each value was obtained by using a stimuli subset consisting of 80% of the stimuli in the original dataset. The higher the value of *J*_*separation*_, the stronger is the discrimination power of the corresponding protein pair.

#### Finite element modeling

In order to calculate the material parameters after the mechanical tests, finite element modeling (FEM) using the software FEBio [24] was used. The cartilage disc was modelled as an axisymmetric 1° wedge element and an unconfined compression scenario was set up. The bottom surface was fixed in the axial direction and sliding contact was set between the top surface of the cartilage element and a rigid compression plane. A bi-phasic material model was chosen. The matrix consisted of a nearly incompressible hyperelastic Neo-Hookean material with an ellipsoidal fiber distribution [25]. Hydraulic permeability was chosen to be strain dependent with the formulation from Holmes-Mow [26]. The top and bottom surfaces were set as impermeable and zero fluid pressure was set at the radial surface of the cartilage wedge.

The same deformation as measured with the mechanical tests was applied at the compression plane, the resulting force was computed and material parameter optimization was performed by minimizing the objective function *f(a)* according to Eq (5) with the Levenberg-Marquardt algorithm using the optimization functionalities of FEBio.

$$f(a)=\sum_{i=1}^{n}{\left[y_{i}-y(x_{i};a)\right]}^{2}$$ (5)

Here, the *(x*_*i*,_*y*_*i*_*)* are the measurement data and *y(x;a)* is the function that extracts the corresponding force from the model. At total four material parameters were fitted. The Young’s modulus of the ground matrix *E*, the hydraulic permeability *k*_*0*_, and the fiber distribution parameters *β* and *ξ*. For more information the reader is referred to the theory manual of the FEBio software suite.

#### Statistical comparisons of individual treatments

Statistical comparisons between individual treatments were done with the unpaired two-sided t-test at significance level p = 0.05.

Table 1 provides an overview of the sample and replicate number for all experiments performed in the study. Furthermore the tissue source (patients P1-P4), the replicate number and the location of the associated results presented in this work are listed.

**Table 1:** Summary of experiments.

Experiment and number of replicates	Patient	Result location
Preliminary analysis: Steady state (n = 2 per tissue state)	P2	S2 File
Preliminary analysis: Batch effect and CV (n = 1 per patient)	P1 + P2	S3 File
Protein release after 3 different collagenase II concentrations (n = 2 per group)	P2	Main text
Healthy and degrading tissue response (n = 1 per tissue state)	P2 + P3	Main text
Histology (n = 2 per tissue state)	P4	Main text
GAG release (n = 5 per tissue state)	P4	Main text
Collagen II content (n = 5 per tissue state)	P4	Main text
Mechanical test (n = 5 per tissue state)	P4	Main text

Overview of the experiments and replicate number per tissue state for all experiments performed in the study, the patient number and the result location.

## Results

### Collagenase II treatment induces OA like phenotype changes

The results of GAG release and collagen II content, mechanical tests and histology of cartilage discs treated for 24h with DMEM* or collagenase II are presented herein.

#### GAG release and collagen II content

**Fig 2:**
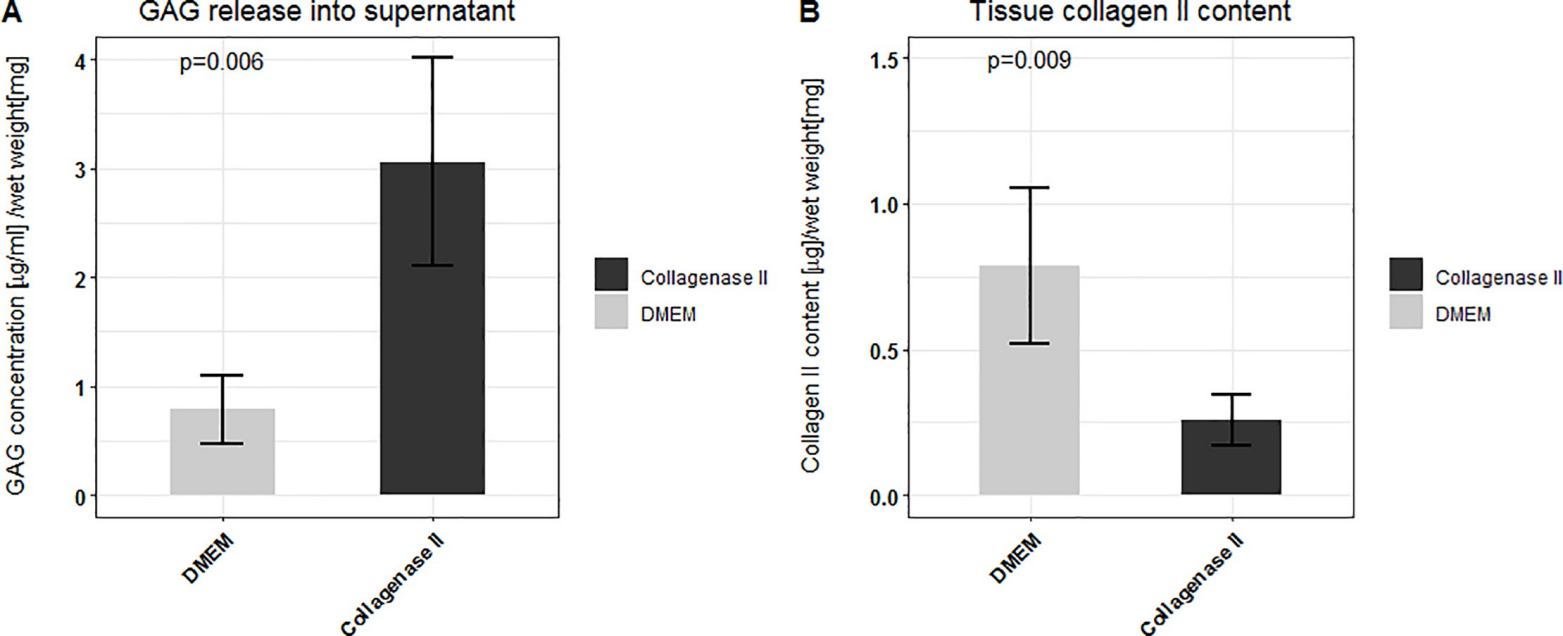
GAG release and collagen II content. A: GAG concentration per mg of tissue wet weight in the supernatant of cartilage samples treated with collagenase II or DMEM* for 24h. B: Tissue collagen II content per mg of tissue wet weight of cartilage samples treated with collagenase II or DMEM* treated samples for 24h. Mean values (n = 5) and standard deviations are shown.

Fig 2 shows the GAG concentration in the supernatant and the tissue collagen II content of DMEM and collagenase II treated samples. It can be observed that collagenase II treatment increases the GAG release into explant supernatant (p = 0.006) and decreases the collagen II content of the tissue (p = 0.009).

#### Mechanical tests and material parameters

The fitted material parameter values are presented in Table 2 with the mean and standard deviation. The asterisk shows statistically significant difference between the DMEM* and the collagenase II group. The Young’s modulus *E* is significantly lower in the collagenase II group (p = 0.0024) and the hydraulic permeability *k*_*0*_ is significantly higher in the collagenase II group (p = 0.006). There are no differences for the fiber distribution parameters *β* and *ξ*.

**Table 2:** Fitted material parameters.

Condition	E*	k_0_*	β	ξ
DMEM*	2.7 +/- 0.6	3e-4 +/- 3e-4	2.3 +/- 0.16	2.7 +/- 0.16
**Collagenase II**	0.8 +/- 0.6	1.8e-3 +/- 5.6e-4	2.7 +/- 0.16	2.7 +/- 0.21

Fitted material parameters after compression tests shown with mean and standard deviation (n = 5). The asterisk (*) marks a statistically significant difference between the DMEM* and the collagenase II group of the respective parameters.

#### Histology

**Fig 3:**
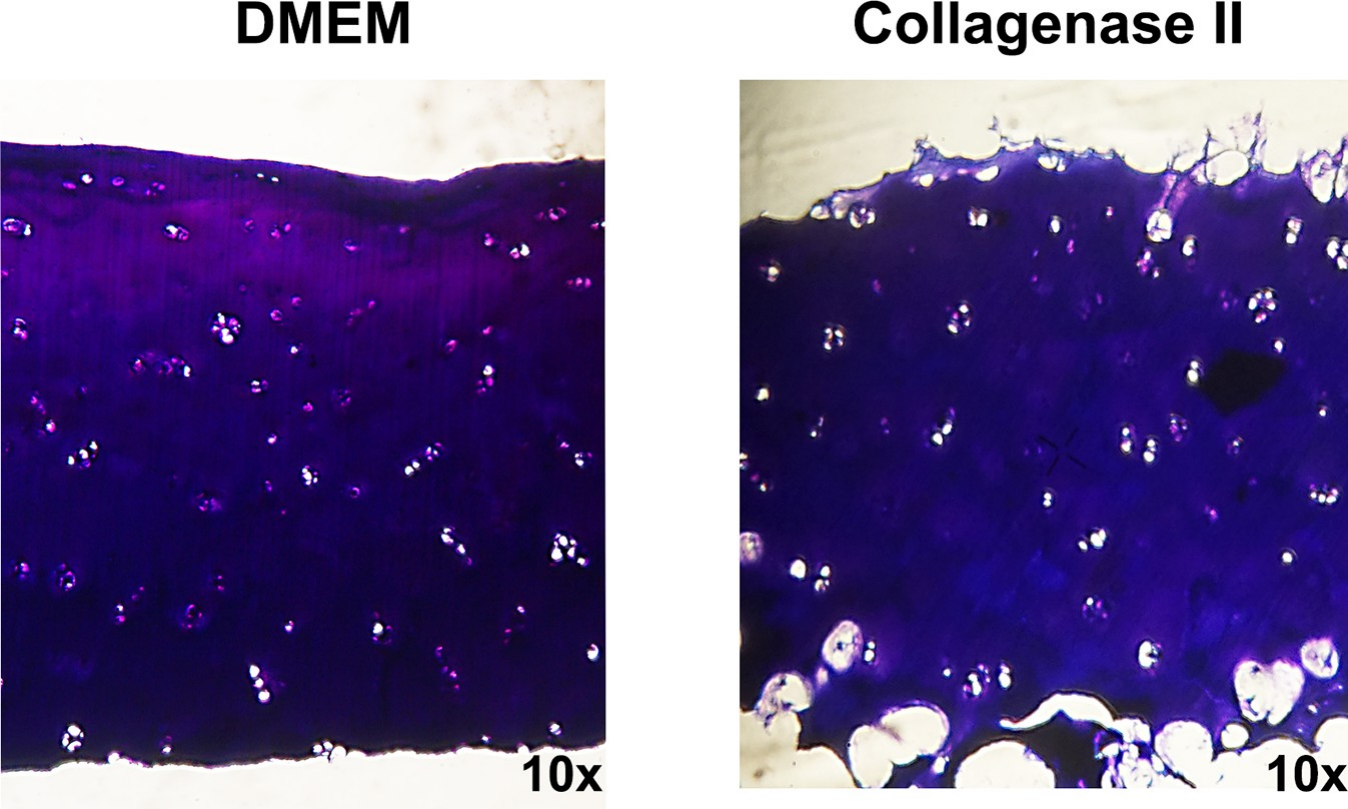
Histological sections of cartilage samples. Representative micrographs (10x) of toluidine blue staining of DMEM* (left) and collagenase II (right) treated cartilage discs.

Fig 3 shows the comparison between DMEM* and collagenase II treated cartilage discs. Surface fibrillation and fissuring is seen in the collagenase treated cartilage. It can be observed that all surfaces of the collagenase II treated samples get degraded.

In summary collagenase II treatment leads to structural changes of the tissue. The material parameters get deteriorated with a decrease of matrix stiffness and an increase of the hydraulic permeability. Further on GAG release is increased and collagen II content is decreased in collagenase II treated samples. Taken together, these results partly confirm the results reported by Grenier et al.[10], in general showing that collagenase II treatment induces OA like phenotype changes.

### Collagenase II treatment induces protein releases observed in clinical OA

In order to investigate the individual protein secretions of collagenase treated tissue, raw MFIs were compared between DMEM* and collagenase II treated samples. The MFIs obtained from the three concentrations were pooled together. Cohen’s d, defined as *d = (m*_*1*_*-m*_*2*_*)/s* where *m*_*i*_ are the class means (i = 1: collagenase II treated samples, i = 2: DMEM* treated samples) and *s* is the pooled standard deviation of the dataset, was taken as the measurement of the effect size. Thus, a high Cohen’s d means that more protein is released in the collagenase II treated samples. The identified cytokines are presented in Table 3, sorted by decreasing effect size. Out of the 11 proteins with significant differences between control and collagenase II treated samples, 4 were found in many OA related studies. These were TNFa and IFNG that are related to inflammation and innate immunity responses as well as the two anti-inflammatory cytokines IL4 and IL13 [27]. CXCL11 [28], IL17F [29], and TNFSF12 [30] (TWEAK) are also considered contributors of OA. The remaining 4 cytokines TFF3 [31], PEDF [32], CCL5 [33] and S100A6 [34] can be considered underreported players. However every protein has been shown to play a role in at least one study.

**Table 3:** Protein release differences.

Protein	Function/Involvement	p	Cohen’s d
IL4	anti-inflammatory	<0.001	5.7
TFF3	not clearly defined	<0.001	5.2
IFNG	innate immunity	0.018	4.2
PEDF	multiple functions	0.001	-3.2
TNFa	pro-inflammatory	0.001	3.1
CCL5	immunity response	0.007	3.0
IL13	anti-inflammatory	0.002	2.6
S100A6	Ca2+ binding	0.009	2.4
CXCL11	T cell chemoattraction	0.011	2.3
IL17F	pro-inflammatory	0.012	1.4
TNFSF12	pro-inflammatory	0.03	1.3

Protein secretions showing significant differences between DMEM* (n = 3) and collagenase (n = 6) treated samples. Sorted according the effect size measured in terms of Cohen´s d. Protein function and p-values are mentioned.

In summary, these results suggest that cartilage discs treated with collagenase increase the secretion of particular proteins that also have been observed to have increased levels in clinical OA studies.

### Tissue state can be determined based on measured protein patterns

**Fig 4:**
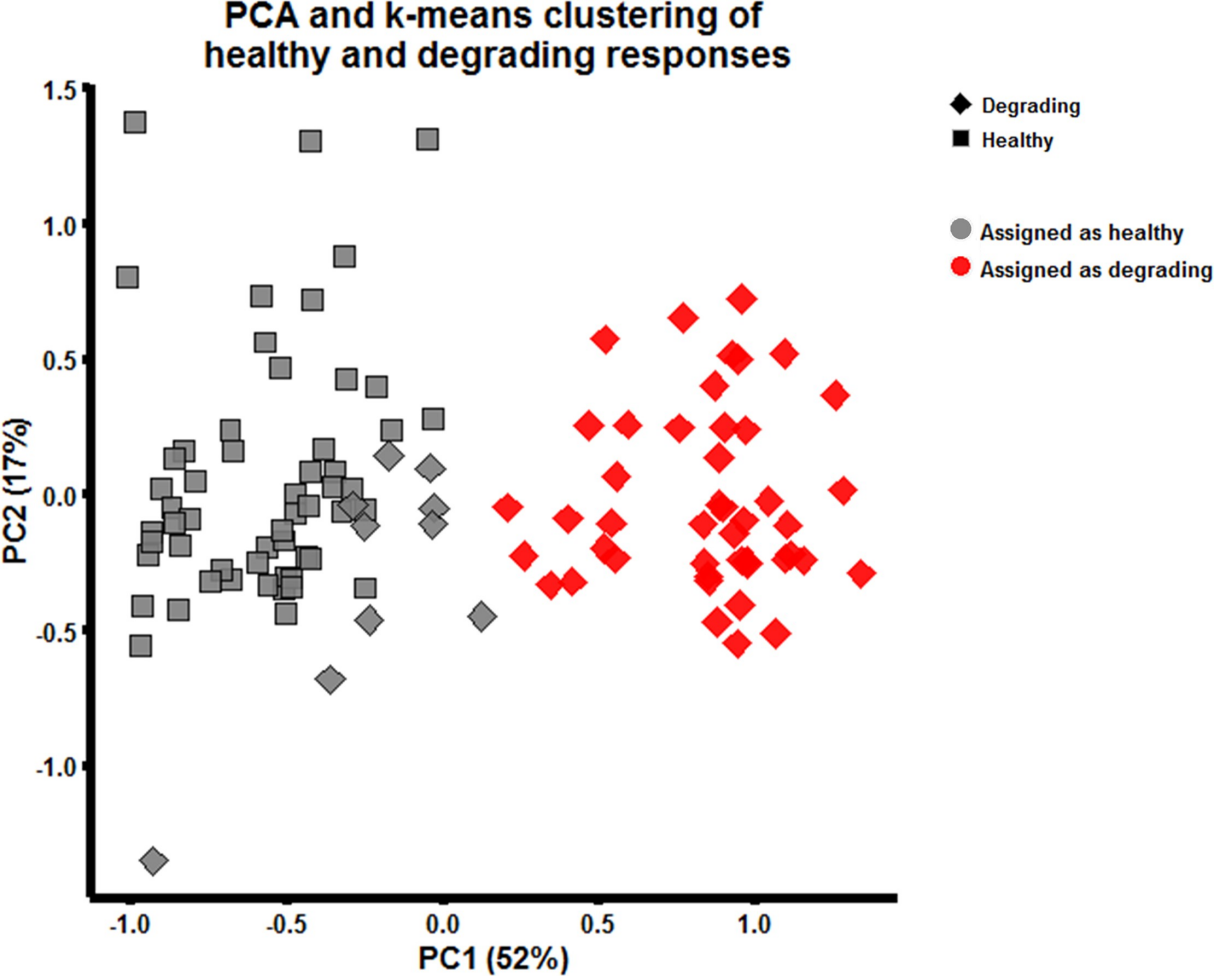
PCA and k-means clustering of tissue responses. PCA score plot (first two principal components covering 52% and 17% of the variance, respectively) of normalized MFIs. The shape of a marker indicates its true tissue group. The colors reflect the unsupervised category assignments made by k-means clustering (k = 2) category assignments. As the samples assigned to the gray color mainly belong to the healthy group, it has been given the label “assigned as healthy” while the red samples has been given the label”assigned as degrading”.

PCA of healthy and degrading tissue responses with subsequent k-means clustering (k = 2) based on the first 4 PCA dimensions (82% of variance covered) were performed. The responses plotted in the resulting 2D space of the first two PCs are presented in Fig 4.

The shapes of the markers in Fig 4 represent the true tissue (sample) groups. There are 55 squares (healthy perturbed discs) and 55 diamonds (degrading perturbed discs) in the PC1-PC2 plane. The two colors gray and red represent the two categories identified by the k-means algorithm (k = 2). The subsequent manual assignment of them as “assigned as healthy” and “assigned as degrading” protein secretion patterns was based on the fact that the majority of the members of the gray cluster belongs to responses from the healthy tissue and the majority of the red cluster belongs to responses the degrading tissue.

Fig 4 also shows that protein secretions after perturbations lead to the formation of two distinct clusters in the PC1-PC2 plane, indicating that tissue state can be determined based on measured protein patterns. In particular, the coordinate along the PC1-axis seems suitable for classification of the cytokine responses as “healthy” or “degrading”. This suggests that it is interesting to look at the elements (loadings) of the corresponding eigenvector in order to determine which of the measured protein changes are most useful for separation between healthy and degrading tissue. The individual loadings of each protein to PC1 are shown in S4 File. Increases of MMP9 and FGF2 as well as decreases of CXCL10, ZG16 and FST would cause a shift along PC1 from degrading to healthy responses.

### Changes in IFNG and MMP9 release after stimulation are promising for tissue state discrimination

**Fig 5:**
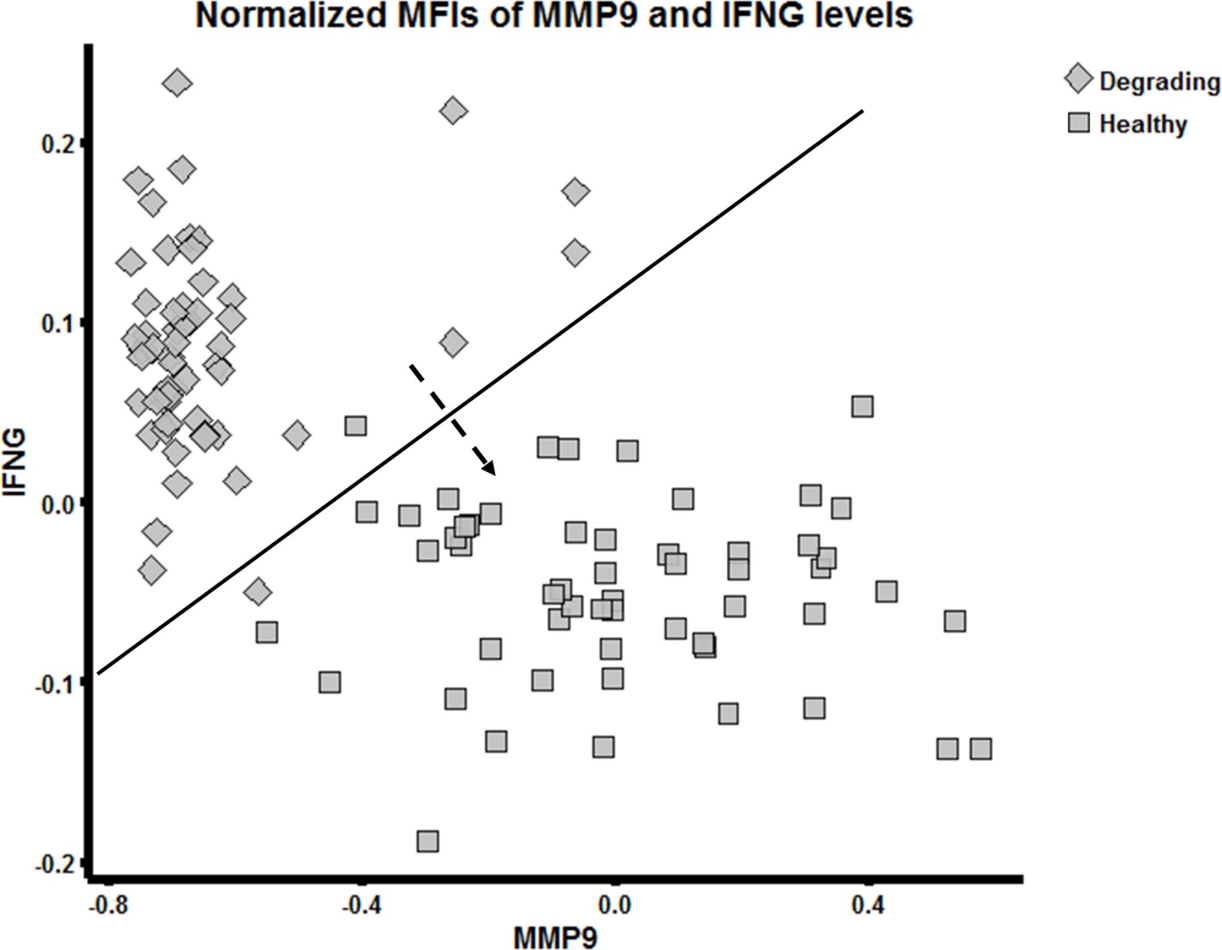
Changes in MMP9 and IFNG release of healthy and degrading tissue due to stimulation. A scatterplot of changed IFNG and MMP9 release (normalized MFIs) in healthy and degrading cartilage. The line indicates a separating linear decision boundary between healthy and degrading tissue. The arrow indicates the desired direction that the degrading responses should move towards.

PCA is an unsupervised method that disregards any class information. Therefore it is not guaranteed that the resulting linear projection provides an optimal solution in terms of discrimination between healthy and degrading tissues. Notably, 10 out of the 55 degrading tissue responses get assigned to the wrong cluster using this approach (Fig 4, gray diamonds). Therefore, supervised discriminant analysis in the form of OOS-DA [20] was used instead, to identify the proteins most accountable for the changes between degrading and healthy responses. The full analysis can be found in S4 File. Briefly, applying OOS-DA identified that there were many linear combinations of the 26 measured protein changes that lead to better separation than using the corresponding PCA projections. Contrary to the analysis of the PC1 loadings, no outstanding influence of FGF2 on class separation could be observed using OOS-DA. Therefore, instead of performing dimensionality reduction using OSS-DA that yields ambiguous results, a straightforward variable subset selection procedure was employed in the form of an exhaustive search across all plausible subsets of two proteins where the optimal protein pair regarding class discrimination was identified. The resulting scatterplot of this pair (MMP9, IFNG) together with a linear decision boundary separating the two classes is shown in Fig 5. The arrow indicates the direction in which the degrading tissue responses should be moved in order to coincide with the healthy tissue responses.

In summary stimulation of healthy and degrading tissue with a set of 55 stimuli lead to changed secretion levels of various proteins, which were evaluated with respect to their ability to discriminate between the two tissue states using different approaches. An exhaustive search across all pairwise combinations of changed protein response levels identified MMP9 and IFNG, among several other pairs, as promising for class discrimination.

## Discussion

The main aim of this study was to measure and characterize cytokine/protein release patterns in an ex vivo model of cartilage degradation, created by exposing healthy cartilage to the ECM degrading enzyme collagenase type II. First, it was shown that collagenase II treatment of healthy cartilage samples leads to OA like phenotype changes. Then it was shown that the induced ECM degradation resulted in baseline secretion patterns of proteins related to OA observed in various clinical studies (Table 3). Further on, the changes in protein release of healthy and degrading tissue due to a broad set of OA related protein perturbations were investigated. Multiplexed ELISA measurements of 26 secreted proteins were subject to exploratory (PCA, k-means) multivariate data analysis. These analyses showed that it is possible to successfully distinguish between healthy and degrading samples for a majority of the samples (Fig 4), but 10 out of the 55 responses from the degrading tissue were assigned incorrectly. Follow-up supervised OOS-DA showed that there are many other linear projections, other than those obtained using PCA (see S4 File), which give almost perfect separation for all samples. Therefore, an exhaustive variable subset selection algorithm was finally employed to identify pairs of proteins that give the most promising discrimination between the classes. The changed levels of MMP9 and IFNG delivered the best distinction between the classes according to the separation criterion used (Fig 5). Notably, the cloud of squares in the scatter plot (Fig 5) may be interpreted as a characteristic distribution (“fingerprint”) showing how a healthy cartilage responds to a panel of 55 protein perturbations. Similarly, the cloud of diamonds in Fig 5 reflects how a degraded cartilage responds to the same perturbations. Ideally a successful drug treatment would make the degraded cartilage produce the same cloud as the healthy cartilage, meaning that the cloud of diamonds moves to sit on top of the cloud of squares, as indicated by the striped arrow in Fig 5.

The low response levels of MMP9 in degrading cartilage is an observation that adds new pieces to the currently unclear picture of the role of MMP9 in this context. More specifically, Fig 5 shows that when the degraded cells are stimulated, MMP9 release drops whereas the opposite happens for healthy cells. Notably this observation does not provide any information regarding if MMP9 has a low release or not compared to healthy cells in the unstimulated state. The question regarding if there is a higher/lower baseline release of MMP9 in degraded cells compared to healthy cells in the unstimulated case is addressed in connection to Table 3 (in fact showing that MMP9 does not have a significantly different baseline release in degrading cartilage compared to healthy). MMP9 (Gelatinase B,92 kDa, type IV collagenase) is known to dissolve ECM and initiate vessel formation [35] and has been shown to play a role in OA. However, ambivalent results exist regarding MMP9 presence in OA joints. For example, the clinical study by Naito et al. showed reduced baseline MMP9 levels in the serum of female knee OA patients [36] whereas the study by Masuhara et al. [37] showed increased baseline MMP9 levels in the serum and plasma of patients with rapidly destructive hip OA. As pointed out in the review on gelatinases in OA [35], the behaviour of these proteins is rather complicated with many interactions and regulatory mechanisms that still need to be understood.

In many studies on in vitro models for OA related drug treatment, the typical experimental readouts are glycosaminoglycan (GAG) and collagen II content. In addition prototypic biomarkers such as matrix metalloproteinases (MMPs) or inflammatory factors such as IL1a/b or TNFa are also often used [38]. Looking at such few readouts might be too simplistic given the outstanding complexity of the human biological systems. In particular, this might lead to misleading conclusions in general, for example overlooking truly efficient single drugs and drug combinations. Therefore, in our approach we measured the changed release of 26 proteins simultaneously. Then we used the collected data in order to characterize the molecular processes associated with CD, including how they can be used for diagnosis and/or to accelerate drug discovery and development.

Both our molecular and phenotypic results support the idea of Grenier et al.[10] to use collagenase II to induce an “OA-like” state. In their work, Grenier et al. performed collagenase II treatment for 45–120 min applied at the upper surface of the samples, and claimed it to induce similarities with early stages of OA [10]. In our work the collagenase II treatment was extended to cover 24h at all sides of the sample. As can be seen in S2 File 6h of treatment with collagenase II does not lead to a steady state level of synthesized and released proteins. Moreover 24h of collagenase II treatment from all sides of the samples is necessary to reach an approximate steady state, therefore using exactly the similar approach as Grenier et al., by treating only the superficial zone for up to 120 min will most probably not result in satisfying measurements.

The panel of released proteins after such a treatment in our study (Table 3) indicates similarities with late rather than early stages of OA, when comparing with clinical studies. However, this difference in interpretation may simply be due to the fact that there is yet no clearly defined distinction between early and late stages of OA.

### Limitations

The systems biology approach introduced here based on protein profiling of CD used 55 stimulations per cartilage condition (healthy and degraded). Since a patient donation usually results in less than 100 cartilage samples, the tissue responses recorded in this study did not come from the same donor. More specifically, healthy perturbed (squares in Fig 4) and degrading perturbed (diamonds in Fig 4) came from two different patients (P2 and P3). Therefore, one cannot exclude that there is a batch effect that can explain some of the differences observed. However, as shown in S3 File, when looking at perturbed healthy cartilage from P1 and P2, no batch effect is observable. Future studies should reduce the number of perturbations employed in each experiment, thereby allowing for using cartilage samples from the same patient for both states (healthy and degrading) on the same 96-well plate. A reduced number of perturbations would also decrease the necessary cartilage material needed per patient as several donors did not have enough cartilage to create the required number of samples. Additionally, using duplicate measurements on the same experimental plate would also be preferable. The relatively high number of 55 different perturbations was used to increase the probability to observe different protein releases from healthy and degrading tissue, whilst accepting a statistically weaker significance of the biological differences observed. Thus, the framework and results presented here should be considered as a proof-of-concept that will be followed by more optimized experiments in the future, which will be limited to a smaller set of stimulations.

In distinction from the in vitro model of Grenier et al. [10], collagenase II treatment was applied at all sample surfaces instead of just the top surface which might be contradictory to the common assumption for OA development and progression at the cartilage superficial zone. As can also be seen in the histological sections in Fig 3 ECM degradation is also observable at the bottom sides of the samples, the same phenomenon was also observed at the lateral sides. As mentioned above, we strongly believe that although treatment of only the superficial zone of the cartilage explants has a higher relevance with respect to OA it will not produce appropriate results in terms of protein releases in order to further investigate the release patterns. There is further on a possibility of bacterial collagenase alternating directly the state of the chondrocytes, however we think that this phenomenon is rather unlikely as bacterial collagenase expresses receptor proteins with high affinity to components of mammalian ECM [39] and should not bind to other cells, e.g. chondrocytes.

Despite these limitations we believe that the novel systemic ex vivo and in silico approach introduced here presents a viable way to investigate compound treatments for CD in general, and related to OA in particular. Thus, we have shown that a more sophisticated systemic analysis at the molecular level is feasible, and that it provides a more detailed molecular picture of what happens during CD in terms of proteomic responses to cytokine/protein stimulations.

## Conclusions

In this study we presented an ex vivo model of cartilage degradation for systemic profiling of interactions between stimulating and responding proteins. Measurements of key protein patterns seem useful to make statements about the condition of an unclassified tissue sample. Future work will investigate changes in tissue responses after pre-treatments with chemical agents (drug candidates) that have the potential to inhibit ongoing cartilage degradation.

## Supporting information

S1 Table Normalized MFIs. Normalized fluorescence intensities of the Luminex experiments of healthy and degrading tissue perturbed with 55 stimuli.
(XLSX)

S1 File Experimental layout. Plate layout and stimuli concentrations for the perturbation experiments.
(PDF)

S2 File Steady state analysis. Measurement of protein releases after 6h, 24h, 48h and 72h of stimulation.
(PDF)

S3 File Analysis of patient-to-patient and assay variation. Batch effect analysis with patients P1 and P2. Analysis of the coefficient of variation per protein in the multiplex assay.
(PDF)

S4 File Further analysis of tissue responses. Loadings of PC1 and detailed presentation of optimal orthonormal system for discriminant analysis.
(PDF)
