# Supplementary material for: An ex vivo tissue model of cartilage degradation suggests that cartilage state can be determined from secreted key protein patterns
Source: PLoS One. 2019 Oct 21;14(10):e0224231. doi: 10.1371/journal.pone.0224231 (PMC6802827; doi:10.1371/journal.pone.0224231)
Supplement: S1 File — Plate layout and stimuli concentrations for the perturbation experiments. (PDF) [file pone.0224231.s002.pdf]

Supporting Information 1

Experimental layout

The experimental layout for stimulation of tissue discs together with the applied concentrations is shown in Figure S1. The bold borders describe the plate, each cell contains a cartilage sample. 130 µl of the top row and the left column were used in order to create 55 (+1 control) stimuli of final concentrations half of the shown values.

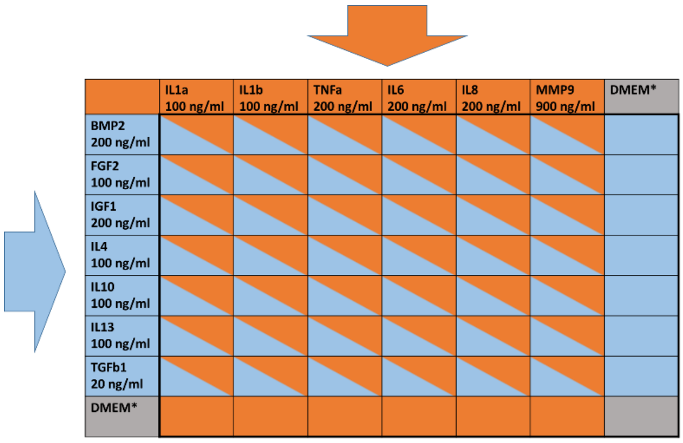

Figure S1: Layout for tissue stimulation. Top row and left column are mixed with an equal amount of 130 µl in order to produce 55 different stimuli.

The stimuli were chosen because of their major and well-known involvement in osteoarthritis. Table 1 describes their function with the respective reference.

Name	Full name and function	Ref.
IL1a	major pro-inflammatory cytokine	[1]
IL1b	major pro-inflammatory cytokine	[1]
TNFa	major pro-inflammatory cytokine	[1]
IL6	dual role/regulatory: pro-inflammatory cytokine and anti-inflammatory potential	[1]
IL8	dual role/regulatory: pro-inflammatory cytokine and anti-inflammatory potential	[1]
MMP9	gelatinase, degrades extracellular matrix	[2]
BMP2	chondrogenic - major role in development of bone and cartilage	[1]
FGF2	growth factor – supports tissue growth in many tissues	[1]
IGF1	growth factor - supports tissue growth in many tissues	[1]
IL4	major anti-inflammatory cytokine	[1]
Il10	major anti-inflammatory cytokine	[1]
IL13	major anti-inflammatory cytokine	[1]
TGFb1	major growth factor	[1]

[1] Mary B Goldring. Osteoarthritis and cartilage: the role of cytokines. *Current rheumatology reports*, 2(6):459–465, 2000.
[2] Brandon J Rose and David L Kooyman. A tale of two joints: the role of matrix metalloproteases in cartilage biology. *Disease markers*, 2016, 2016.
